# Resilience to climate-induced disasters and its overall impact on well-being in Southern Africa: a mixed-methods systematic review protocol

**DOI:** 10.1186/s13643-018-0796-4

**Published:** 2018-08-21

**Authors:** Joseph K. Kamara, Nidhi Wali, Kingsley Agho, Andre M. N. Renzaho

**Affiliations:** 1 School of Social Sciences and Psychology, Western Sydney University, Sydney, NSW Australia; 2 World Vision International, Southern Africa Regional Office, Mbabane, Swaziland; 3 School of Science and Health, Western Sydney University, Sydney, NSW Australia

**Keywords:** Disasters, Resilience, Well-being, Communities, Southern Africa

## Abstract

**Background:**

Southern Africa has long been vulnerable to climate-induced disasters, especially droughts and floods. The severity and frequency of disasters increased in the early 1980s, continuously eroding livelihoods, which in turn invoked humanitarian intervention. A systematic review of the relationship between resilience to drought and well-being will be undertaken.

**Methods:**

Studies will be included if they were conducted between January 1980 and December 2017; used quantitative and/or qualitative methods; were peer reviewed or comprise grey literature; covered Southern Africa; and measured resilience and its relationship to well-being. Data extraction will be informed by the Cochrane Public Health Group and the Joanna Briggs Institute manuals. The quality of evidence of the studies included will be assessed for risk bias, psychometric properties of tools used, and their suitability. The findings will be summarised into themes and narrated.

**Discussion:**

This protocol is guided by the Preferred Reporting Items for Systematic Reviews and Meta-Analyses Protocols (PRISMA-P) guidelines. The protocol gives insight of the scope and parameters for the systematic review to be carried out. The systematic review will establish how resilience to climate-induced disasters affects well-being. It will also provide recommendations to improve humanitarian coordination in Southern Africa.

**Systematic review registration:**

The protocol was registered by the PROSPERO international prospective register of systematic reviews, reference CRD42017064396.

**Electronic supplementary material:**

The online version of this article (10.1186/s13643-018-0796-4) contains supplementary material, which is available to authorized users.

## Background

Globally, disasters are increasingly becoming more frequent and omnipresent. A disaster is a calamity that results in injury, loss of life, as well as social and economic disruption that exceeds the coping capacity of its victims [1]. Some disasters are manmade while others occur naturally. Man-made disasters result from human action such as wars, pollution, industrial accidents, and environmental degradation. Natural disasters are the slow or rapid onset of climatological (drought, heatwave, wild fires), geophysical (volcanoes, earthquakes, tsunamis), hydrological (floods, avalanches), meteorological (storms, cyclones, heatwaves), and biological (epidemics, plagues, or insects) occurrences [2].

Humans have limited control over natural disasters but have the inherent ability to respond and adapt. The ability to respond and adapt is progressively making resilience to natural disasters an important area of interest. For many reasons, our systematic review will focus on resilience to climate-induced drought in Southern Africa. Firstly, the region is one of the most affected by natural disasters. [3–5]. Literature suggests that Southern Africa has been vulnerable to climate-induced disasters since the early 1900s [4–6]. However, the 1980s saw an increase in the frequency, severity, and intensity of disasters, particularly droughts [7–10]. Most of the drought occurrences necessitated humanitarian intervention to avert intra-regional crises. Some studies have argued that Southern Africa, which is highly vulnerable to disasters, remains poorly prepared to respond due to high environmental degradation, weak social-economic status, poor governance, and high poverty levels [8, 9]. Secondly, while other sub-Saharan African regions are also affected by disasters, there are differences in the context and nature of responses. Southern African countries have different development trajectories from other sub-Saharan African regions. Southern Africa, for example, has the highest number of middle-income countries compared to the rest of the continent. Finally, there are differences in donor bias compared to the rest of sub-Saharan Africa, with natural disasters, such as drought, attracting higher and quicker donor funding in East or West Africa than in Southern Africa [11–14]. This affects timely disaster response. These contextual differences are significant and may negatively affect the external validity of the findings of any study seeking to look at the effect of resilience to climate-induced drought on well-being across sub-Saharan Africa. Restricting our analysis to Southern Africa will provide region-specific findings that will inform regional policies and strategies to improve the utility and policy translation of the findings.

The governments overseeing the region have not prioritised strengthening resilience to cyclical drought-induced disasters, which has often led to a reactive disposition that undermines development gains [10]. Responding to disasters imposes a high burden on economies. Most of the governments in the region have resorted to the externalisation of disaster response to the humanitarian community as a coping mechanism [15]. It is apparent that governments require assistance when their resources are overwhelmed by sudden disaster occurrences as a result of which the humanitarian community is urged to help. However, governments have a mutual obligation to build and strengthen resilience, especially to repetitive threats such as droughts, which have become a ‘new normal’.

In this region, focused attention also needs to be given to reducing the dependence on maize, the staple food, which is widely grown by way of rain-fed farming, highly intolerant to weather changes, and one of the key vulnerabilities to drought [16]. Notwithstanding the weak capacities and vulnerabilities, there is a gradual shift in rhetoric from reactive disaster response and recovery operations to proactively building resilience [13]. Nonetheless, the region’s governments are yet to work out a comprehensive approach to address droughts. Droughts are ‘slow onset’ and subject to creep, with extended impact that outlasts the drought itself. The severity of the aftermath poses the criticality of facilitating measures of resilience to drought for continuity, well-being, and sustainability of affected communities. However, resilience remains poorly understood and is often overlooked in periods of crisis in Southern Africa.

Resilience is a broad subject without a generally accepted definition. Various scholars have defined resilience differently within the contexts of their research, and research questions have sought to address resilience to what, by whom, and how. This implies setting specific parameters for exploring the subject. For our systematic review, resilience is seen as the ability to harness one’s environment to anticipate, endure, recover from, and adapt to disaster-induced distress [17]. Such ability occurs at individual, household, and collective level. People do not live in isolation of their families and communities. Therefore, the application of resilience behaviours and qualities depends on the collective individual, family, community systems as well as assets. Our systematic review will incorporate individual household (family) and, collectively, community resilience coping mechanisms. This approach will allow the assessment of collective action for resilience (individual, family, and community efforts) as well as the communal life of the study area. We anticipate outcomes that will be contextually and culturally appropriate for the region.

For the purpose of this study, we define well-being as the state of people’s healthiness, comfort, delight and the “… realisation of their potential” [18], p8. Simply stated, well-being is the absence of ill health. Southern Africa is the southernmost region of Africa and comprises Botswana, Lesotho, Namibia, South Africa, and Swaziland as categorised by the United Nations regional grouping [19].

## Aim of the review and its public health significance

The aim of the review is threefold: (i) examine the relationship between resilience to drought and well-being, (ii) examine the suitability of the resilience instruments in studies retained for review and examine their psychometric properties, and (iii) identify gaps and unanswered questions for further inquiry. Available literature suggests that resilience to disasters may be a determinant of health [20, 21]. However, there has been no attempt to synthesise evidence of the relationship between disasters, such as droughts and well-being, especially among the rural communities in Southern Africa. Understanding the relationship between disasters, especially drought resilience and well-being, and the ways affected communities cope, will not only influence policies but will also improve the coordination of humanitarian action in the region. This systematic review is necessary to appraise, synthesise, summarise, and refine literature on resilience and well-being in the region frequently affected by droughts. Diverse literature, including policy documents, working papers, and organisation reports, will be included to ensure the review captures advances made in policy. Therefore, the review is important for informing intervention strategies and policies as well as knowledge enhancement.

## The review question

The systematic review will be guided by the following question: is there a relationship between resilience and well-being among the drought affected communities?

## Methods and design

### Types of studies targeted, inclusion, and exclusion criteria

This protocol is informed by the standard Preferred Reporting Items for Systematic Reviews and Meta-Analyses Protocols (PRISMA-P) reporting guidelines [22]. The PRISMA-P checklist is attached to this manuscript as Additional file 1. Both peer-reviewed and grey literature on disaster resilience and well-being in Southern Africa Region will be included. The following criteria will be used for inclusion:

- Peer-reviewed articles, dissertations, books and book chapters, working papers, technical reports, discussion papers, and conference papers published between January 1980 and December 2017:
- Studies written in English and whose full texts are available and accessible. The research team does not have the financial and logistical capacity to retrieve and translate articles published in languages other than English;
- Studies which measured resilience and its relationship to health and well-being.

The reference lists of studies included will be scanned for relevant articles that meet the inclusion criteria. Authors and organisations identified in the reference lists of studies will be contacted to obtain their work that may be referred to but which is unavailable. A search log will be kept for accountability and transparency.

Studies will be excluded if they

- Were published outside the stated time frame or conducted in countries outside the Southern Africa region;
- Were published in languages other than English;
- Are reviews, editorials, letters to editors, and opinion pieces; and/or
- Did not assess the relationship between resilience and drought well-being.

The year 1980 has been selected as the base for the search for many reasons. It marked a period of the intensification of climate-induced disasters in Southern Africa. For example, strong El Nino occurrences began to occur at more frequent intervals of less than 2 years resulting into low food production, and food and water crises [23, 24]. Furthermore, the 1980s denoted improved disaster governance, such as the establishment of the inter-government agency known as the Southern Africa Development Community (SADC). The SADC was established to “achieve development and economic growth, alleviate poverty, enhance the standard and quality of life of the people of Southern Africa and support the socially disadvantaged through regional integration” [25], p7. Other notable changes during the 1980s were the introduction of the drought aid scheme for farmers, investment, and refurbishment of the region’s transport system to ease the movement of goods and services, and the introduction of the famine early warning system (FEWS NET) in drought monitoring [26–29]. The period also marked the end of the first green revolution, which produced a variety of maize, cassava, millet, and sorghum suitable for the African context to prevent hunger and food insecurity [28, 30–32].

### Types of participants

There will be no limits to the age, gender, social status, or ethnicity of participants. The setting of studies included will be rural communities in Southern Africa. A large proportion of the regional population subsists on agriculture, is rural-based, and is increasingly faced with food shortages [13, 33, 34].

### Outcomes of interest

The outcomes of interest include individual and family resilience, disaster preparedness and response, psychological well-being, social support, and neighbourhood cohesion. Other key outcomes of interest include any measures of resilience and measures of its relationship to well-being. We will seek to understand resilience as an outcome when defined as ability to withstand and recover from a disaster event [35]. Thus, all outcomes, including unintended ones, reported in studies that meet the inclusion criteria will be captured in our review.

### Search strategy

The systematic review will be reported in accordance with the Preferred Reporting Items for Systematic Reviews and Meta-Analyses (PRISMA) guidelines. The search will apply appropriate search terms and subject heading truncations (*), and Boolean operators depending on the specifications of databases to be searched. Various databases for peer-reviewed and grey literature will be searched using subject heading truncations and search terms on resilience and well-being in the disaster-prone Southern Africa. The appropriate search query has been developed and tested to search in PubMed on 5 September 2017, as reflected in Additional file 2.

### Databases

To ensure a wide catchment of relevant content, the following electronic databases will be searched: African Journals Online (AJOL), Applied Social Sciences Index, CINAHL, Environmental Complete, EMBASE, MEDLINE, Scopus, Social Science ProQuest Central, PsycINFO, Web of Science and Wiley Online Library. Additionally, the search syntax will be adapted to meet multidisciplinary databases that may require visual inspection of individual records for potential relevance. These are Global Health Library, and key organisation websites, especially Africa Development Bank, DFID, FAO, OECD, USAID, and the World Bank.

### Data extraction

Two researchers JKK and NW will sift records for inclusion and code their decisions within their EndNote Library following a 3-staged screening process. Foremost, studies will be screened by title to eliminate duplicates, followed by screening of the remaining studies’ abstracts for eligibility and relevance. Lastly, full texts of articles judged eligible from the abstract screening will be obtained and reviewed. Where uncertainty emerges, at any stage, a third reviewer (AR) will be consulted to adjudicate eligibility. A piloted form based on the Cochrane Public Health Group manual will be used in the extraction of quantitative studies [36]. The extraction of qualitative studies will be based on a form adopted from material by the Joanna Briggs Institute [37]. The data to be extracted will include country of study, year of publication, study objective, design and setting, sampling and data collection method, theory and/or hypothesis, resilience outcome measures, including instrument strength and well-being, and a comments section.

### Quality assessment

The quality assessment of studies included will be independently undertaken by JKK and NW. It will involve two different analyses: risk bias and instrument psychometric properties. Risk bias analysis will involve exploration of limitations and appropriateness of study methods in addressing their research questions and objectives, and how they inform outcomes. Particularly, studies will be critically assessed for their design, data collection and analysis methods, selection bias, integrity, confounders, attrition, and reporting. Thereafter, we will categorise and summarise the findings uncertain, high, or low [38]. The Cochrane Collaboration tool for risk bias will be used in the assessment of controlled trials [39]. ROBINIS-I tool will be used to assess all other quantitative studies such as non-controlled trials and quasi-experiments [40]. The dependability of qualitative studies will be appraised with a piloted form adopted from the Joanna Briggs Institute Qualitative Assessment and Review Instrument [37].

The psychometric strength of instruments will be assessed for reliability and validity (criterion, construct and content) of the scales using the framework by Cyril and colleagues [41]. The reliability of the instruments will be assessed for internal consistency and test–retest reliability [38, 42]. For internal consistency, Cronbach’s alpha will be used for scoring as follows: < 0.50 = unacceptable (0 point), > = 0.50 and < 0.70 = poor (1 point), > = 70 and < 0.80 = acceptable (2 points), and > = 0.80 = good (3 points). For test–retest reliability, the intra-class correlation coefficient or Kappa will be used for scoring as follows: < 0.40 = poor. (0 points), > = 0.40 and < 0.60 = fair (1 point), >= 0.60 and < 0.75 = good (2 points) and > = 75 = good (3 points). Criterion validity will determine the correlation between resilience scales and well-being outcomes. This will examine the correlation indices between resilience scores with wellbeing as follows: 0 no linear relationship (0 point); 0.30 = a weak linear relationship (1 point); 0.50 = a moderate relationship (2 points); > = 0.70 = a strong linear relationship (3 points). Construct validity will assess whether the scales used were actually measuring resilience and detect structure in the relationships between resilience subscales. This will be assessed by examining whether the scales were derived by means of exploratory and confirmatory factor analyses, and whether extracted subscales met minimum threshold for a factor analysis. The following criteria will be used for scoring. Extracted factors explained > = 50% of the variance (yes = 1 point, no = 0 point), each extracted factor has at least 3 items (yes = 1 point, no = 0 point), each variable loads strongly on only one factor (> = 0.35) and has two or more strong loadings (> = 0.70) (yes = 1 point and no = 0 point), and the factor analysis was based on at least 10 cases per variable (yes = 1 point and no = 0 point). Content validity will examine whether all aspects of resilience had been taken into consideration [43]. This will involve examining how items were elected and whether the target population and a panel of experts were involved in item selection. The scoring will use the following criteria: item development informed by a literature review or empirical study (yes = 1 point or no = 0 point), was reviewed by the target population (yes = 1 point and no = 0 point), and reviewed used panel of experts (yes = 1 point, no = 0 point). The total psychometric properties scoring will range between 0 and 13 points: 0–4 points = poor; 4–9 points acceptable, and 10–13 points very good. Any scoring or rating disagreements that emerge between JKK and NW will be addressed through a kappa statistic.

### Data analysis

Due to the heterogeneity and variation of the studies to be reviewed—especially the study methods, measurements, and outcomes—a statistical aggregation of the data may not be appropriate. Instead, a narrative synthesis, based on tables of ratings and frequencies will be undertaken [41–44]. Common threads and trends will be identified and extracted from both qualitative themes and quantitative narratives to generate insight on resilience programming in drought situations. All study results will be aggregated to provide a holistic analysis. However, the conclusion to be made will be based on studies scoring high on quality. JKK will summarise the study findings and narrate the emerging themes. NW will review the appropriateness of the content as well as the consistency of the emerging themes.

## Discussion

There is increasing interest among governments, the donor community, and humanitarians in Southern Africa to understand and promote disaster resilience as a long-term strategy to mitigate the devastating effect of recurring disasters [10, 13, 45, 46]. The devastating impact of drought and the need to understand resilience make it a research and policy priority. Ample literature exists on the subject matter but remains unsynthesised. The vast literature supports the need for a systematic review to provide a robust summary of evidence that can be drawn upon to influence drought management policies and commensurate planning and interventions. Additionally, the existing literature does not address the relationship between resilience and well-being in disaster-prone rural Southern African communities. This review will use well-known and validated tools to synthesise evidence from the substantial pool of literature, to identify gaps, and to frame a way forward that will improve our understanding of the relationship between resilience to climate-induced disasters, especially drought and well-being. The findings of the systematic review will inform policies and strategies to combat the effect of drought and improve humanitarian coordination in the region.

We anticipate some limitations, such as the exclusion of studies published in other languages than English. Exclusion of such studies could lead to missing key literature generated by non-English-speaking researchers, especially those within the study population who might have a unique perspective on the subject matter. Nonetheless, the review will explore a range of literature to capture and include as many studies as possible to overcome the limitation.

## Additional files

Additional file 1: PRISMAP checklist. (DOCX 37 kb)

Additional file 2: Piloted search query. (DOCX 14 kb)

## Abbreviations

AJOL: African Journals Online
CINAHL: Cumulative Index to Nursing and Allied Health Literature
DFID: Department of International Development (United Kingdom Government)
EMBASE: Excerpta Medica DataBASE
FAO: Food and Agriculture Organisation (of the United Nations)
FEWS NET: Famine Early Warning System Network
MEDLINE: Medical Literature and Retrieval System Online
OECD: Organisation for Economic Co-operation and Development
PRISMA: Preferred Reporting Items for Systematic Reviews and Meta-Analyses
PRISMA-P: Preferred Reporting Items for Systematic Reviews and Meta-Analyses Protocols
PROSPERO: International Prospective Register of Systematic Reviews
ROBINIS-I: Risk of Bias in Non-Randomised Studies-of Interventions
SADC: Southern Africa Development Community
USAID: United States Agency for International Development
