# Supplementary material for: Resilience to climate-induced disasters and its overall impact on well-being in Southern Africa: a mixed-methods systematic review protocol
Source: Syst Rev. 2018 Aug 21;7:127. doi: 10.1186/s13643-018-0796-4 (PMC6103869; doi:10.1186/s13643-018-0796-4)
Supplement: Supplementary file 2 — Piloted search query. (DOCX 14 kb) [file 13643_2018_796_MOESM2_ESM.docx]

**Piloted search query with key search terms and their combination to be used**

| **#** | **Key Search Terms** |
| --- | --- |
| 1 | (Resilien* OR psychological resilien* OR adapt* OR adaptive behavio* OR behavio* OR coping behavio* OR coping* OR skill* OR coping skill* OR adjustmen* OR social adjustmen* OR coheren* OR coherence sense*) |
| 2 | (Well-being* OR wellbeing* OR Health* OR disparit* OR status disparit* OR status indicat* OR indicato* OR health status indicato* OR index* OR health status index* OR indice* OR health status inde* OR metric* OR metric system*) |
| 3 | (Southern Africa* OR southern* OR region* or southern region*OR Botswana* OR Lesotho* OR Namibia* OR Swaziland* OR South Africa* OR Africa*) |
| 4 | #1 AND #2 AND #3 Filters: Full text; Publication date from 1980/01/01 to 2017/09/30; English |
